# Construction of a neural network diagnostic model for renal fibrosis and investigation of immune infiltration characteristics

**DOI:** 10.3389/fimmu.2023.1183088

**Published:** 2023-06-09

**Authors:** Yangyang Guo, Kenan Cen, Kai Hong, Yifeng Mai, Minghui Jiang

**Affiliations:** ^1^ Department of General Surgery, The First Affiliated Hospital of Ningbo University, Ningbo, China; ^2^ Department of Urology Surgery, Taizhou Hospital of Zhejiang Province Affiliated to Wenzhou Medical University, Taizhou, Zhejiang, China

**Keywords:** renal fibrosis, machine learning, immune cell, CIBERSORT, diagnostic biomarker

## Abstract

**Background:**

Recently, the incidence rate of renal fibrosis has been increasing worldwide, greatly increasing the burden on society. However, the diagnostic and therapeutic tools available for the disease are insufficient, necessitating the screening of potential biomarkers to predict renal fibrosis.

**Methods:**

Using the Gene Expression Omnibus (GEO) database, we obtained two gene array datasets (GSE76882 and GSE22459) from patients with renal fibrosis and healthy individuals. We identified differentially expressed genes (DEGs) between renal fibrosis and normal tissues and analyzed possible diagnostic biomarkers using machine learning. The diagnostic effect of the candidate markers was evaluated using receiver operating characteristic (ROC) curves and verified their expression using Reverse transcription quantitative polymerase chain reaction (RT-qPCR). The CIBERSORT algorithm was used to determine the proportions of 22 types of immune cells in patients with renal fibrosis, and the correlation between biomarker expression and the proportion of immune cells was studied. Finally, we developed an artificial neural network model of renal fibrosis.

**Results:**

Four candidate genes namely DOCK2, SLC1A3, SOX9 and TARP were identified as biomarkers of renal fibrosis, with the area under the ROC curve (AUC) values higher than 0.75. Next, we verified the expression of these genes by RT-qPCR. Subsequently, we revealed the potential disorder of immune cells in the renal fibrosis group through CIBERSORT analysis and found that immune cells were highly correlated with the expression of candidate markers.

**Conclusion:**

DOCK2, SLC1A3, SOX9, and TARP were identified as potential diagnostic genes for renal fibrosis, and the most relevant immune cells were identified. Our findings provide potential biomarkers for the diagnosis of renal fibrosis.

## Introduction

Renal fibrosis refers to the proliferation of fibrous tissue in the kidney due to trauma, infection, inflammation, and other factors, resulting in a gradual deterioration of renal function (1). Its pathological characteristics include damage to intrinsic renal cells, stimulation of the inflammatory response, activation of fibroblasts and myofibroblasts, accretion of extracellular matrix (collagen fibers, fibronectin, and laminin), loss of intrinsic renal cells, atrophy and collapse of renal tubules, and thinning of blood vessels, which ultimately result in damage to the kidney structure (2). Renal fibrosis mainly includes glomerulosclerosis, tubulointerstitial fibrosis, and intrarenal vascular sclerosis. The diagnosis of renal fibrosis depends on renal biopsy, which is invasive and prone to bleeding (3). Therefore, it is important to identify easily available and specific biomarkers of renal fibrosis.

Recently, comprehensive bioinformatics analysis based on high-throughput sequencing has been used to screen new prognostic biomarkers related to a variety of diseases (4). Zhou et al. found that Wnt-induced secreted protein-1 (WISP-1) is increased in animals with renal fibrosis and may provide a new target for the treatment of renal fibrosis (5). Sun et al. revealed that ISG20 knockout significantly inhibits the progression of renal fibrosis *in vitro*, indicating that ISG20 may play an important role in renal fibrosis (6). Using RNA sequencing analysis, Shuo et al. revealed that Gal-3 was highly expressed in renal fibrosis biopsy samples and positively correlated with the severity of renal fibrosis, which supported the effect of Gal-3 in predicting renal fibrosis (7). However, the exploration of a joint diagnostic model of multiple genes for renal fibrosis is still insufficient.

Therefore, our research aimed to analyze the differential expression of genes between renal fibrosis disease and healthy individuals, screen diagnostic markers for renal fibrosis and determine the relationship between biomarkers, immune cell levels, and drug sensitivity.

## Methods

### Data acquisition

The gene expression levels in renal fibrosis and control samples were obtained from the gene expression omnibus GEO database. The dataset GSE76882 was used as the training set, which included 99 controls and 175 renal fibrosis samples. All samples were standardized for subsequent analyses. To verify the reliability of the neural network model, the dataset GSE22459 was used as the validation set, which included 25 control samples and 40 renal fibrosis samples.

### Differential expression of genes screening, weighted correlation network analysis and enrichment analysis

The different expression of genes (DEGs) between the control samples and renal fibrosis samples were screened using the R package “limma” The screening conditions were: log fold change (FC) was greater than 2 and false discovery rate (fdr) was less than 0.05. Subsequently, we obtained the module with the highest correlation with renal fibrosis through weighted correlation network analysis (WGCNA) (8) and obtained renal fibrosis-related DEGs through the intersection. Next, we used the R package “clusterProfiler”, “org. Hs. eg. db” and “DOSE” to conduct gene ontology (GO) and the Kyoto Encyclopedia of Genes and Genomes (KEGG) enrichment (9, 10).

### Identification and verification of predictive markers for renal fibrosis

Least absolute shrinkage and selection operator (LASSO) logistic regression and support vector machine-recursive feature elimination (SVM-RFE), were used to identify predictive genes for renal fibrosis (11–15). The R package “glmnet” was used for LASSO analysis, and the optimal variable was found by the SVM-RFE algorithm. Candidate diagnostic markers were screened using these two algorithms and verified using reverse transcription quantitative polymerase chain reaction (RT-qPCR).

### Correlation analysis between immune cells and candidate biomarkers

Immune cells in renal fibrosis and control samples were evaluated using the CIBERSORT algorithm. Spearman rank correlation analysis was performed using the R package “ggplot2” to visualize the correlation between candidate biomarkers and various immune cells (16).

### The gene set enrichment analysis

GSEA was performed to analyze the potential biological functions of the candidate genes. A gene set named “c2.cp.kegg.v7.0. symbols.gmt” was downloaded from the Molecular Signatures Database (MSigDB) and selected as the reference gene set (17).

### Drug sensitivity analysis

To identify additional drugs targeting the candidate biomarkers for the treatment of renal fibrosis, we conducted a drug sensitivity analysis. The CellMiner database was used to download gene expression data and drug sensitivity data, and the R package “impute”, “limma”, “ggplot2” and “ggpubr” were used for drug sensitivity analysis (18).

### Cell culture and drug treatment

HK-2 cells, a human renal tubular epithelial cell line, were obtained from The American Type Culture Collection (ATCC, Manassas, VA, USA) and maintained in DMEM containing 10% FBS and 1% penicillin-streptomycin at 37°C with 5% CO_2_. In the experimental group, we incubated the cells for 48h with recombinant TGF-β1 (5 ng/ml; PeproTech, USA).

### Reverse transcription-quantitative polymerase chain reaction

Total RNA was extracted using the TRIzol reagent. Total RNA was reverse transcribed into the cDNA template according to the manufacturer’s protocol, and SYBR Green Real-Time PCR Master Mix Plus (Toyobo) was used to amplify the cDNA template. β-Actin was selected as an endogenous reference gene to normalize the mRNA levels. Primer sequences used for RT-qPCR are listed in **Supplementary Table S1**.

### A renal fibrosis classification model established by an artificial neural network

First, the DEG expression data were converted to a Gene Score table based on expression levels. The medians of all the sample expression values and the expression values of a single gene in a given sample were compared. If the expression value of the upregulated gene was greater than 0, it was given a value of 1; otherwise, it was given a value of 0. Likewise, if the expression value of the downregulated gene was higher, it was given a value of 0; otherwise, it was given a value of 1. Renal fibrosis was the outcome variable; cases were assigned a value of 1, whereas controls were assigned a value of 0. An artificial neural network model was visualized using the R package neuralnet (19) based on the constructed Gene Score table. The model parameters were set to five hidden layers. To optimize the model and reduce overfitting, the R package Caret (20) was used to calculate the 5-fold cross-validation of the artificial neural network model.

## Results

### Identification of DEGs and enrichment analysis

**Figure 1:**
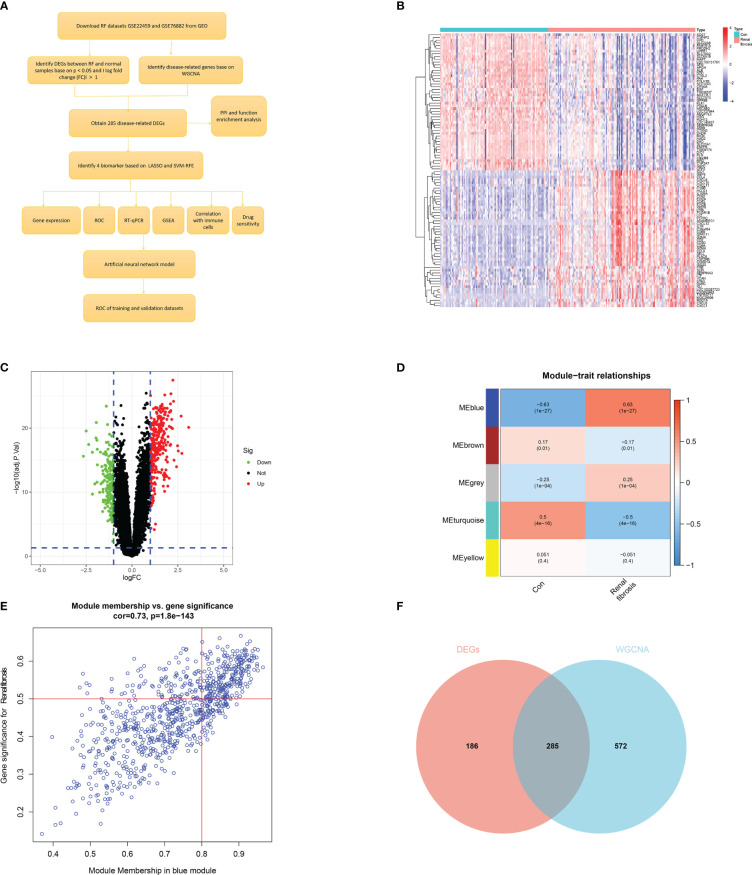
Identification of renal fibrosis-related DEGs. **(A)** Flowchart of the research. **(B)** Heat map of differentially expressed genes between renal fibrosis samples and healthy samples. **(C)** Volcano plot of differentially expressed genes between renal fibrosis samples and healthy samples. **(D)** weighted correlation network analysis of train cohort. **(E)** Correlation between blue module and renal fibrosis. **(F)** Venn of DEGs and WGCNA.

The flow chart of this study was shown in **Figure 1A**. First, we screened 471 differentially expressed genes (DEGs) between the renal fibrosis group and the control group in GSE76882 using the R package “limma” (**Figures 1B, C**). Next, we obtained five modules through WGCNA and found that the blue module had the highest correlation with renal fibrosis (**Figures 1D, E**). We crossed these two results to obtain 285 renal fibrosis-related DEGs (**Figure 1F**).

**Figure 2:**
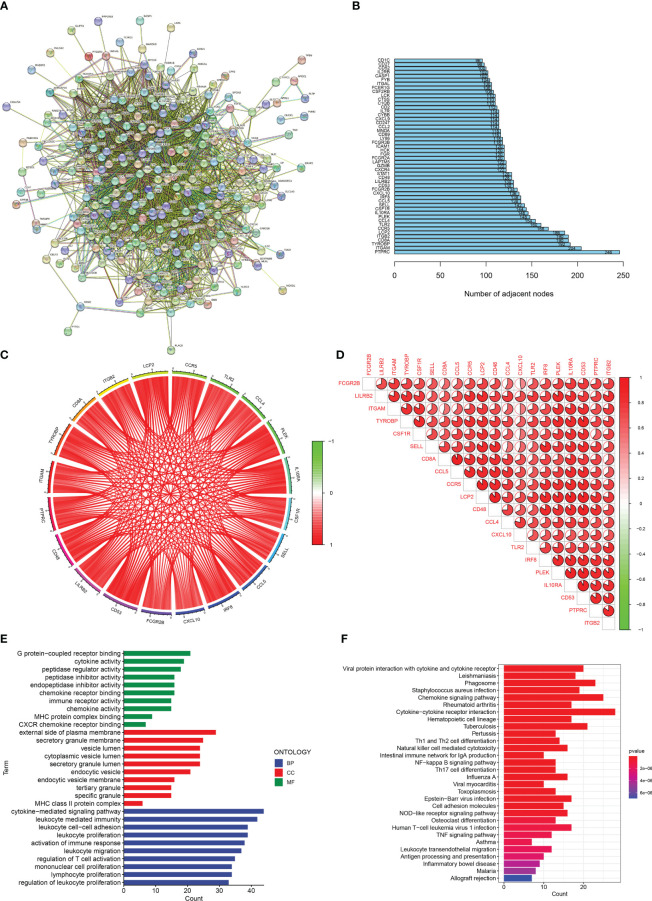
Enrichment analisis of renal fibrosis-related DEGs. **(A)** PPI network of renal fibrosis-related DEGs. **(B)** The number of connection nodes of hub genes. **(C, D)** Correlations between top 20 hub genes. **(E)** The top 10 most significantly enriched GO terms. **(F)** The top 30 most significantly enriched KEGG pathways.

A protein-protein interaction (PPI) network (**Figure 2A**) was constructed for DEGs using STRING (https://cn.string-db.org/). **Figure 2B** shows the number of connection nodes of hub genes. Next, correlation analysis revealed strong positive correlations between these hub genes (**Figures 2C, D**). In addition, we explored the possible biological functions of DEGs using enrichment analysis. The GO enrichment analysis revealed that the DEGs were principally enriched in leukocyte proliferation, MHC class II protein complex, and chemokine activity (**Figure 2E**). KEGG enrichment analysis showed enrichment of multiple immune-related signaling pathways including the chemokine signaling pathway, Th1 and Th2 cell differentiation, intestinal immune network for IgA production, and cytokine-cytokine receptor interaction (**Figure 2F**). These results indicate that immune response may contribute to the occurrence and development of renal fibrosis.

### Identification of the diagnostic markers for renal fibrosis

**Figure 3:**
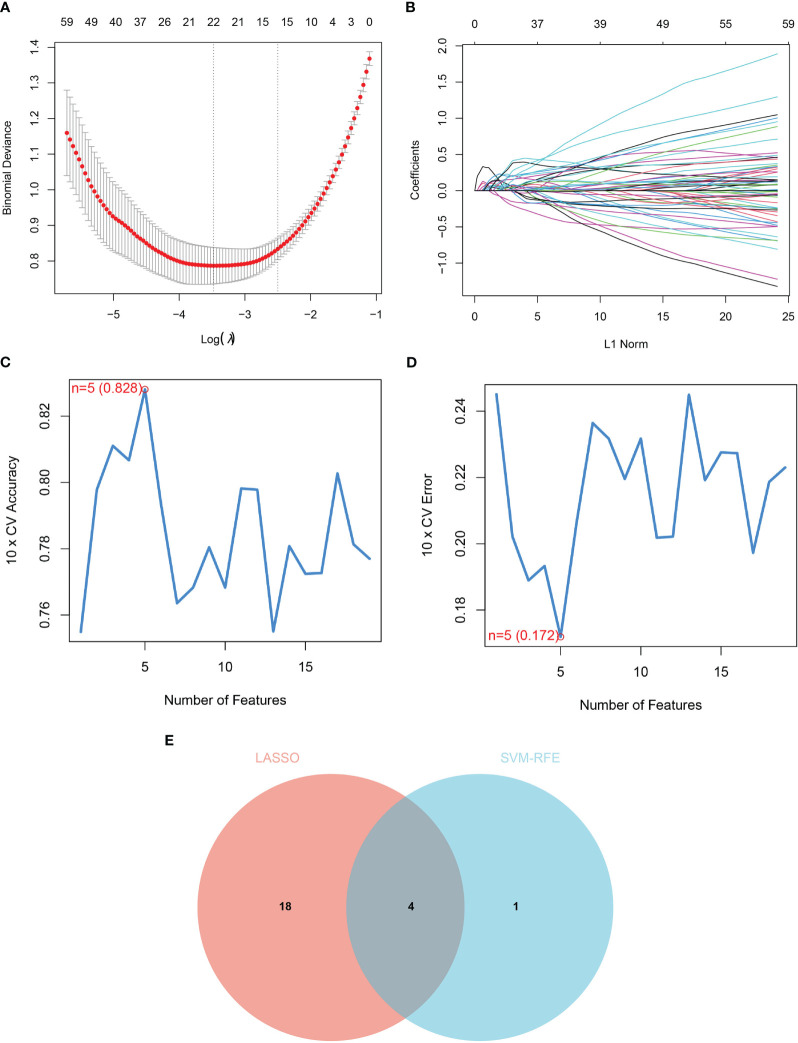
Identification of diagnostic markers for renal fibrosis. **(A, B)** Tuning feature screening in the LASSO model. **(C, D)** A plot of biological marker screening via the SVM-RFE arithmetic. **(E)** Venn graph displaying 4 diagnosis biomarkers shared by LASSO and SVM-RFE.

We used two machine-learning algorithms to identify diagnostic markers of renal fibrosis. First, LASSO regression algorithm was used to reveal 22 potential biomarkers (**Figures 3A, B**). SVM-RFE analysis of the DEGs identified five genes that could be used for diagnosis (**Figures 3C, D**). Next, we determined the common biomarkers obtained in both machine learning algorithms to obtain four common biomarkers: DOCK2, SOX9, SLC1A3, and TARP (**Figure 3E**).

### Diagnostic power and expression of four candidate biomarkers

**Figure 4:**
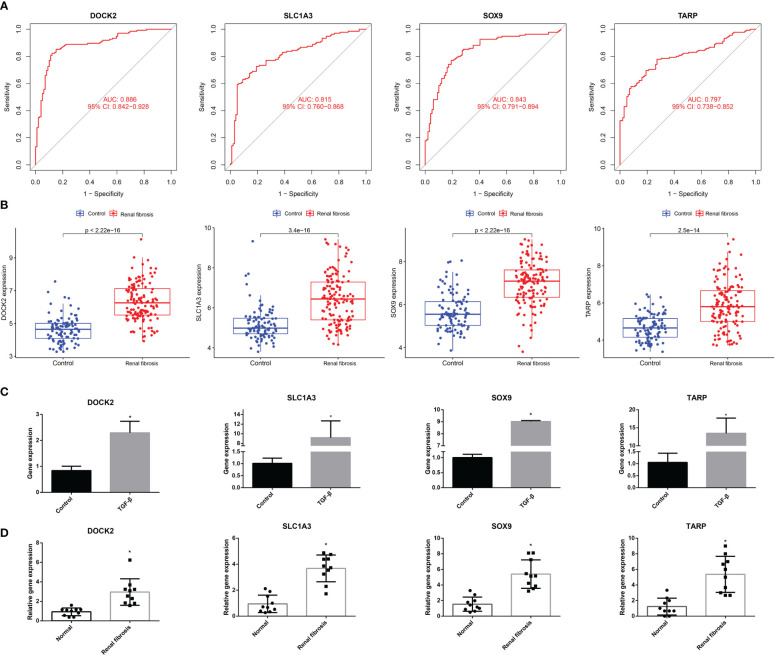
The ROC curve and expression of candidate biomarkers. **(A)** The ROC curve of DOCK2, SOX9, SLC1A3 and TARP. **(B)** The expression of DOCK2, SOX9, SLC1A3 and TARP in GSE76882. **(C)** RT-qPCR of DOCK2, SOX9, SLC1A3 and TARP in HK-2 cells with or without TGF-β. **(D)** The expression of DOCK2, SOX9, SLC1A3 and TARP in patients with renal fibrosis and healthy individuals.

We conducted a comprehensive analysis of the four candidate genes. We plotted receiver operating characteristic (ROC) curves for the four candidate biomarkers and found that DOCK2, SLC1A3, SOX9, and TARP had good diagnostic efficacy, with the area under the ROC curve (AUC) values of 0.886, 0.815, 0.843, and 0.797, respectively (**Figure 4A**).

Next, we estimated the expression of the four candidate diagnostic genes. As shown in **Figure 4B**, the expression levels of DOCK2, SLC1A3, SOX9, and TARP were higher in the renal fibrosis group than in the control group. In addition, we conducted RT-qPCR experiments and discovered that DOCK2, SLC1A3, SOX9, and TARP were also highly expressed in HK-2 cells after TGF-β1 treatment, which was consistent with the results of bioinformatics analysis (**Figure 4C**). We also found the expression of DOCK2, SLC1A3, SOX9, and TARP were higher in blood of renal fibrosis patients than healthy individuals (**Figure 4D**).

### Correlation between candidate biomarkers and immune cells

**Figure 5:**
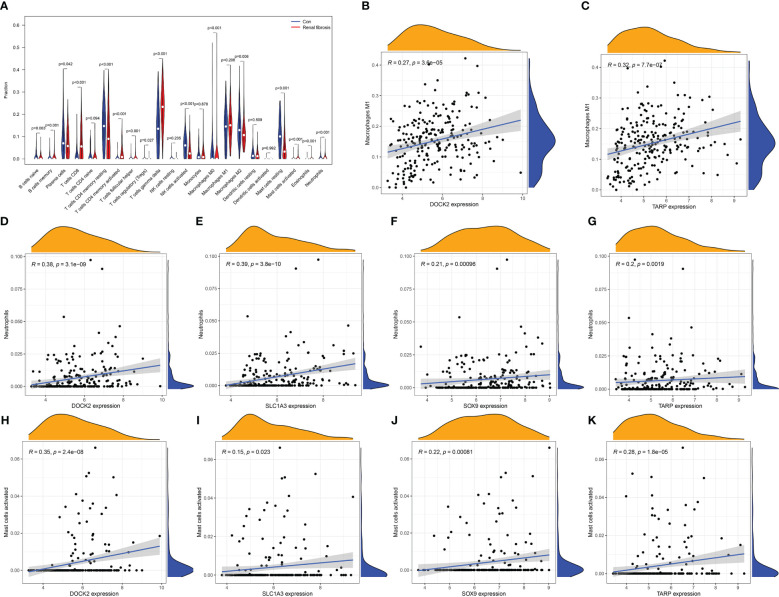
Immune cell infiltration in renal fibrosis samples and control samples. **(A)** Comparison of 22 immune cell types in renal fibrosis samples and control samples. **(B–K)** Correlation between candidate biomarkers and neutrophils, macrophages M1, activated mast cells.

The incidence of renal fibrosis is also accompanied by abnormalities in both the proportion and function of immune cells. Hence, we further analyzed the relationship between the four candidate biomarkers and the 22 immune cells. First, the CIBERSORT algorithm was used to analyze the change in the proportion of immune cells between renal fibrosis and control samples. We found that the ratio of CD8 T cells, activated CD4 memory T cells, follicular helper T cells, gamma delta T cells, macrophages M0, activated mast cells, neutrophils, and eosinophils were increased in the renal fibrosis group, whereas the ratio of plasma cells, resting CD4 memory T cells, T cells regulatory (Tregs), activated NK cells, macrophages M2 and resting mast cells was decreased (**Figure 5A**).

**Figure 6:**
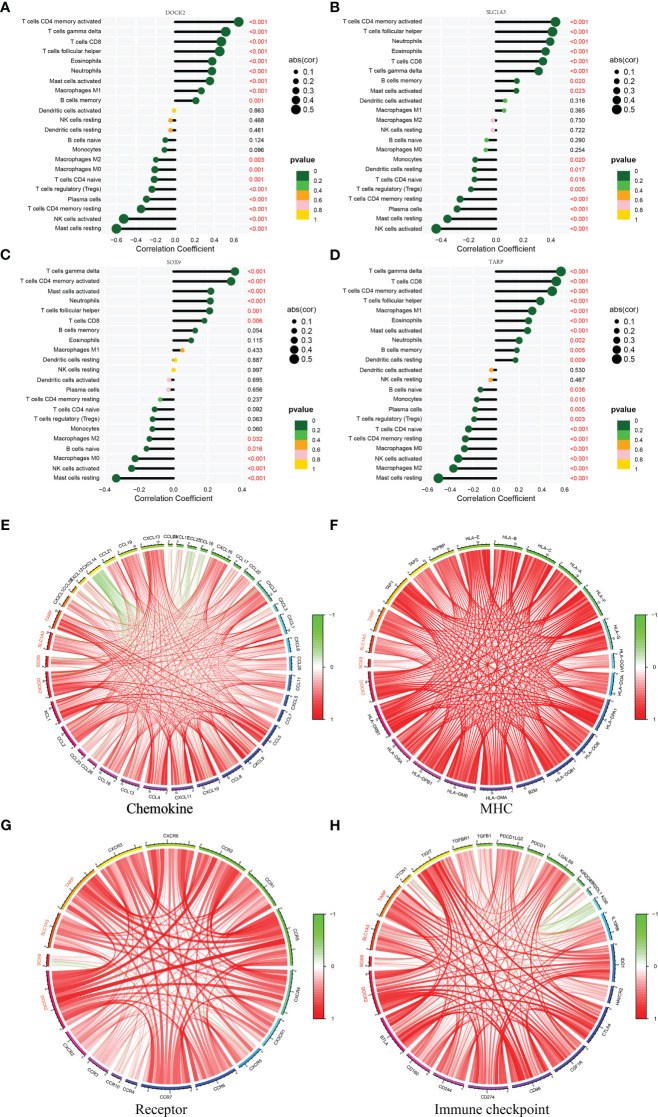
Correlation between four candidate biomarkers and immune cells. **(A)** DOCK2. **(B)** SLC1A3. **(C)** SOX9. **(D)** TARP. Correlation between four candidate biomarkers and immune related genes. **(E)** Chemokine. **(F)** MHC. **(G)** Receptor. **(H)** Immune checkpoint.

In addition, DOCK2 and TARP expression positively correlated with macrophages M1 (**Figures 5B, C**). All candidate biomarkers were positively correlated with activated NK cells and neutrophils (**Figures 5D–K**). Besides, the expression of DOCK2 was positively correlated with activated CD4 memory T cells, gamma delta T cells, CD8 T cells, follicular helper T cells, eosinophils, and memory B cells and negatively correlated with the ratios of macrophages M2, macrophages M0, naive CD4 T cells, Tregs, plasma cells, resting CD4 memory T cells, resting mast cells, and activated NK cells (**Figure 6A**). SLC1A3 expression was positively correlated with the degree of activation of CD4 + memory T cells, follicular helper T cells, Eosinophils, CD8 T cells, gamma delta T cells, and memory B cells, and negatively correlated with monocytes, resting DCs, native CD4 + T cells, Tregs, resting CD4 + memory T cells, plasma cells, resting mast cells, and activated NK cells (**Figure 6B**). SOX9 expression was positively correlated with gamma delta T cells, activated CD4 memory T cells, follicular helper T cells, and CD8 T cells, and negatively correlated with macrophages M2, naive B cells, macrophages M0, resting mast cells, and activated NK cells (**Figure 6C**). TARP expression was positively correlated with activated CD4 memory T cells, follicular helper T cells, resting DCs, Eosinophils, CD8 T cells, gamma delta T cells, and memory B cells, and negatively correlated with monocytes, native CD4 T cells, Tregs, resting CD4 memory T cells, plasma cells, resting mast cells, macrophages M0, macrophages M2 and activated NK cells (**Figure 6D**). In addition, we found that these candidate biomarkers had strong positive correlations with immune-related genes, including chemokines, MHC, receptor and immune checkpoints (**Figures 6E–H**).

### Signaling pathways associated with the candidate biomarkers

**Figure 7:**
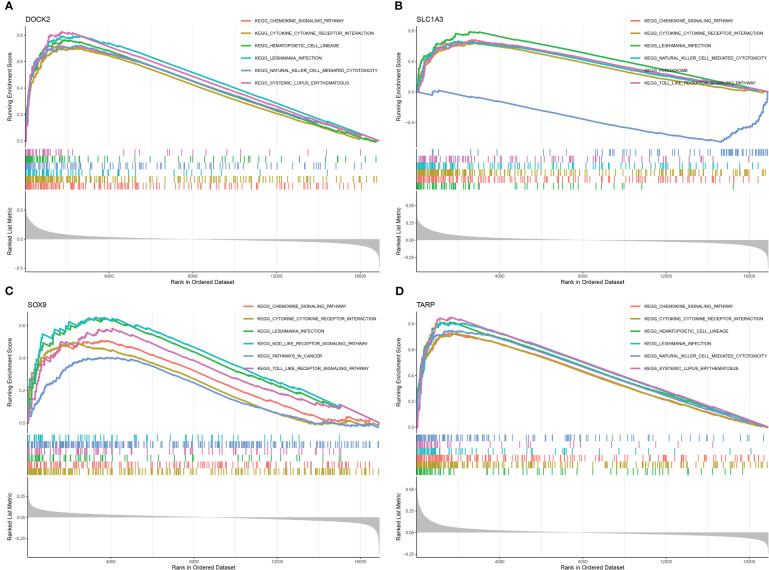
Gene set enrichment analysis (GSEA) identifies signaling pathways involved in the candidate biomarkers. **(A)** DOCK2. **(B)** SLC1A3. **(C)** SOX9. **(D)** TARP.

The relevant signaling pathways associated with the four candidate biomarkers were identified using GSEA. DOCK2 was positively associated with the chemokine signaling pathway, cytokine-cytokine receptor interaction, and Leishmania infection (**Figure 7A**). SLC1A3 mainly participates in Leishmania infection, chemokine signaling pathways, and cytokine-cytokine receptor interactions (**Figure 7B**). SOX9 was positively associated with cytokine-cytokine receptor interactions, chemokine signaling pathways, and toll-like receptor signaling pathways (**Figure 7C**). TARP was positively linked to hematopoietic cell lineage, cytokine-cytokine receptor interaction, and chemokine signaling pathway (**Figure 7D**). These results indicate that the candidate biomarkers were closely correlated with the chemokine signaling pathway, cytokine-cytokine receptor interaction, and Toll-like receptor signaling pathway, which might be instrumental in the pathogenesis and development of renal fibrosis.

### Drug sensitivity analysis

**Figure 8:**
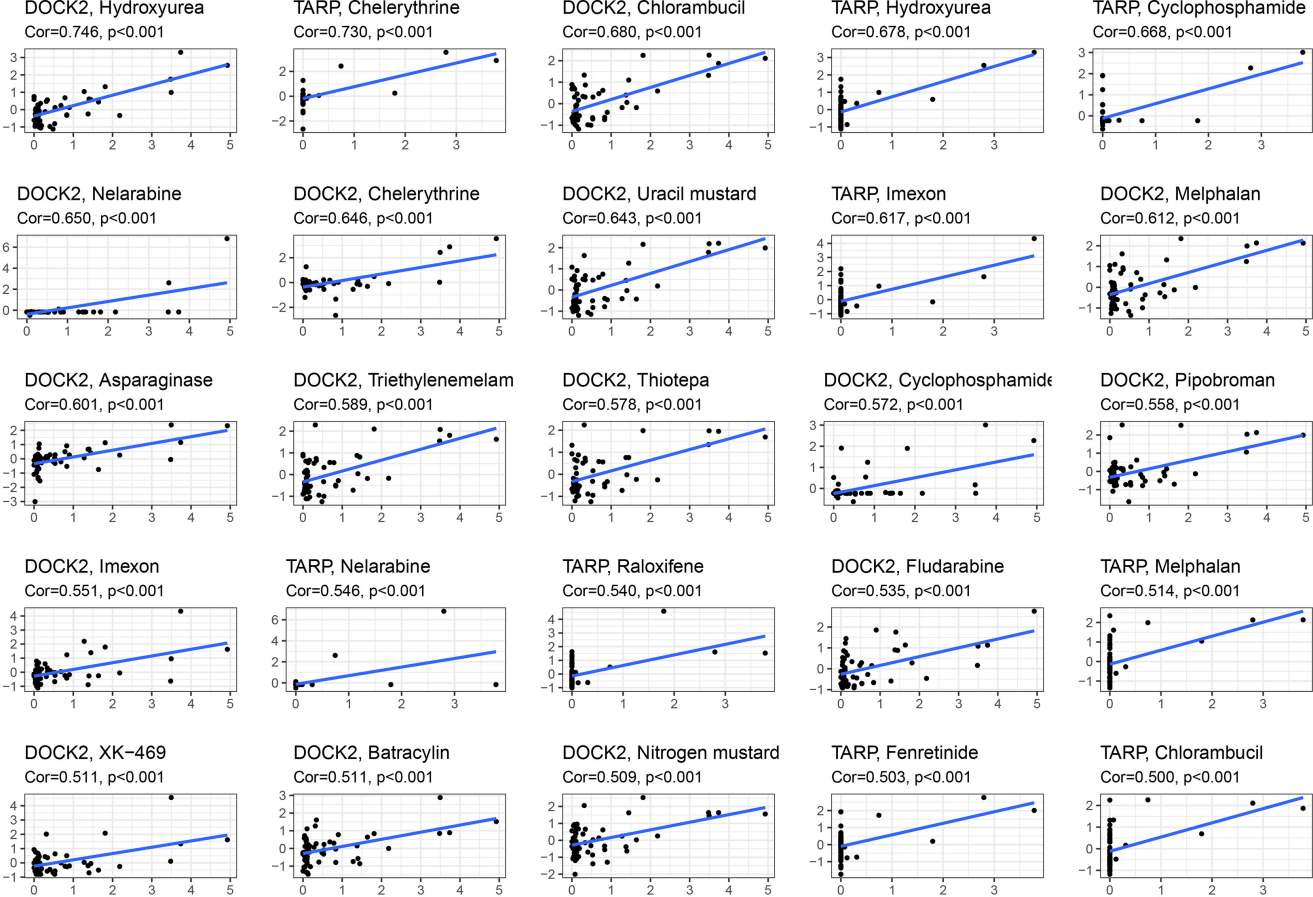
Drug sensitivity analysis of candidate biomarkers.

We performed a drug sensitivity analysis to identify additional drugs that could improve renal fibrosis (**Figure 8**). As a result, the expression of DOCK2 positively linked with Hydroxyurea, Chlorambucil, Nelarabine, Chelerythrine, Uracil mustard, Melphalan, Asparaginase, Triethylenemelamine, Thiotepa, Cyclophosphamide, Pipobroman, Imexon, Fludarabine, XK-469, Batracylin and Nitrogen mustard. The expression of TARP was positively linked to Chelerythrine, Hydroxyurea, Cyclophosphamide, Imexon, Nelarabine, Raloxifene, Melphalan, Fenretinide and Chlorambucil.

### Constructing an artificial neural network model

**Figure 9:**
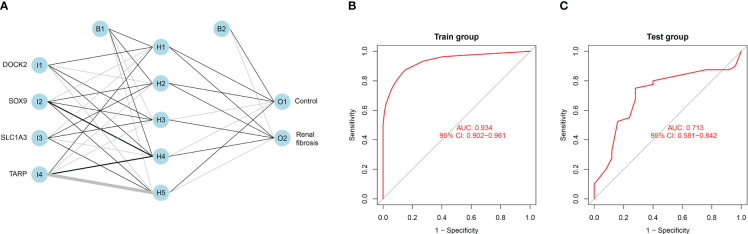
Constructing an artificial neural network model. **(A)** Artificial neural network model for renal fibrosis. **(B)** The training group verifies the ROC curve findings. **(C)** The testing group verifies the ROC curve findings.

We set up a Gene Score table for the renal fibrosis outcome variable (**Supplementary Table S2**). Based on the Gene Score table, we built an artificial neural network model to classify gene expression data. Four input, five hidden, and two output layers were used in the artificial neural network model (**Figure 9A**). The AUC value was 0.934 and its accuracy was 0.874, demonstrating the reliability of the model (**Figure 9B**). To verify the reliability of the model, we analyzed the test group and found that the ROC value was 0.713 (**Figure 9C**).

## Discussion

Currently, renal biopsy remains the primary clinical diagnostic modality for renal fibrosis. Renal biopsy is traumatic, challenging to perform repeatedly, challenging to observe dynamically, and cannot be performed in patients with contraindications (21). Moreover, the renal puncture tissue is less than 1%, mainly cortex, and less medulla, which has limitations in the diagnosis of chronic kidney disease, especially renal fibrosis. Therefore, identifying a noninvasive examination method that can reflect the degree of renal fibrosis and objectively evaluate its efficacy is indispensable. In this study, we screened renal fibrosis biomarkers using machine learning and constructed an artificial neural network model. The identification of these biomarkers will facilitate the diagnosis of renal fibrosis, the selection of therapeutic approaches, and the prediction of treatment responses.

In the present study, we first screened 471 DEGs between the renal fibrosis and control groups and then obtained the renal fibrosis-related module genes. We then combined these results to obtain 285 renal fibrosis-related DEGs. These DEGs were mainly enriched in multiple immune-related signaling pathways, strongly suggesting that the immune response is instrumental in the pathogenesis of renal fibrosis.

Two separate algorithms (LASSO and SVM-RFE) were used to screen for potential diagnostic biomarkers for renal fibrosis. Four genes with overlapping features (DOCK2, SLC1A3, SOX9, and TARP) were finally selected. The roles of these genes in fibrotic diseases have been reported previously. For instance, Xia et al. found that DOCK2 deficiency could weaken bleomycin-induced pulmonary fibrosis through TGF-β signaling pathway (22). Recent research by Gu et al. revealed that the knockdown of SOX9 could reduce injury-induced tracheal fibrosis by inhibiting granulation tissue proliferation, ECM deposition, and inflammatory reactions, and promoting granulation tissue apoptosis (23). Gesine et al. Revealed that deletion of SOX9 in fibroblasts reduced cardiac fibrosis, thereby improving left ventricular dysfunction, dilation, and myocardial scar formation after myocardial infarction (24). In addition, Varinder et al. found that the inactivation of SOX9 in mice prevented fibrosis of the parenchyma and bile and improved liver function and chronic inflammation (25). However, the value and mechanism of action of these biomarkers in renal fibrosis have not yet been clearly studied, which needs to be elucidated in future research.

The chemokine signaling pathway is implicated in renal fibrosis. Renal fibroblasts secrete CCL2, CCL3 and other inflammatory chemokines, which drive various inflammatory cells to the lesion site, thereby promoting the formation of renal interstitial fibrosis (26, 27). A large number of cytokines, such as IL-1, IL-2, IL-6, IL-8, and TGF-β, can be further released by activated macrophages to promote the development of fibrosis (28). In this study, we found that four candidate biomarkers were involved in the chemokine signaling pathway and cytokine-cytokine receptor interactions. Therefore, targeting these signalling pathways may be an effective strategy for the treatment of renal fibrosis.

Renal fibrosis is often accompanied by a series of changes in the proportion and function of immune cells. According to Purvi et al., IL-17 secretion may participate in the pathogenesis of renal fibrosis after AKI via neutrophil recruitment (29). In obstructive nephropathy, GSDMD-dependent neutrophil extracellular traps could accelerate renal fibrosis through macrophage-to-myofibroblast transition (30). Interferon-related factors 4 and 5 are involved in macrophage activation, the release of pro-inflammatory mediators by pro-inflammatory macrophage M1, causing tissue inflammation and renal fibrosis (31). In addition, Kurusu et al. showed that mast cell infiltration per unit volume was positively correlated with the severity of tubulointerstitial fibrosis, suggesting that mast cells are involved in the occurrence and progression of renal fibrosis (32). In this study, we found that four candidate biomarkers were significantly associated with the infiltration of multiple immune cells, including macrophages M1, neutrophils, and mast cells. These results indicate that immune cells participate in the occurrence and development of renal fibrosis, and research on immune cells is an important direction for treating renal fibrosis in the future.

Given that renal fibrosis and cancer share a common feature of enhanced epithelial mesenchymal transition (EMT), as well as the inhibitory effects of many anti-tumor drugs on EMT, we analyzed the relationship between these drug sensitivity and model genes to provide possible direction for the treatment of renal fibrosis. The results showed that DOCK2 and TARP were positively correlated with most drugs. These findings lay the foundation for anti-fibrosis drugs.

Although this study constructed a novel neural network model to predict renal fibrosis based on high-throughput sequencing data, it had several limitations. Firstly, this was a retrospective study with a relatively small sample size, which remains unreliable. Therefore, a prospective study with a larger sample size is warranted.

In summary, we determined four potential diagnostic biomarkers in the peripheral blood of patients with renal fibrosis through machine learning and comprehensively analyzed their diagnostic value, drug sensitivity, and correlation with immune cells. These findings will help improve the diagnosis and treatment of renal fibrosis.

## Data availability statement

The original contributions presented in the study are included in the article/**Supplementary Material**. Further inquiries can be directed to the corresponding author.

## Author contributions

MJ and YG designed and directed the study. KH wrote the manuscript. KC and YM performed data collection and curation. KH performed RT-qPCR experiments. All authors have read and agreed to the published the version of the manuscript. All authors contributed to the article and approved the submitted version.

## References

[1] Wang M Zeng F Ning F Wang Y Zhou S He J. Ceria nanoparticles ameliorate renal fibrosis by modulating the balance between oxidative phosphorylation and aerobic glycolysis. J Nanobiotechnol (2022) 20(1):3. doi: 10.1186/s12951-021-01122-w PMC8725394 34983531

[2] Lv W Booz GW Wang Y Fan F Roman RJ. Inflammation and renal fibrosis: recent developments on key signaling molecules as potential therapeutic targets. Eur J Pharmacol (2018) 820:65–76. doi: 10.1016/j.ejphar.2017.12.016 29229532 PMC6733417

[3] Jiang K Ferguson CM Lerman LO. Noninvasive assessment of renal fibrosis by magnetic resonance imaging and ultrasound techniques. Transl Res (2019) 209:105–20. doi: 10.1016/j.trsl.2019.02.009 PMC6553637 31082371

[4] Nguyen ND Wang D. Multiview learning for understanding functional multiomics. PloS Comput Biol (2020) 16(4):e1007677. doi: 10.1371/journal.pcbi.1007677 32240163 PMC7117667

[5] Zhou Y Chai P Wang J Li L Chen MH. Wingless/int-1induced secreted protein-1: a new biomarker for renal fibrosis. J Biol Regul Homeost Agents (2021) 35(1):97–103. doi: 10.23812/20-459-A 33480221

[6] Sun YC Qiu ZZ Wen FL Yin JQ Zhou H. Revealing potential diagnostic gene biomarkers associated with immune infiltration in patients with renal fibrosis based on machine learning analysis. J Immunol Res (2022) 2022:3027200. doi: 10.1155/2022/3027200 35497880 PMC9045970

[7] Ou SM Tsai MT Chen HY Li FA Tseng WC Lee KH. Identification of galectin-3 as potential biomarkers for renal fibrosis by RNA-sequencing and clinicopathologic findings of kidney biopsy. Front Med (Lausanne) (2021) 8:748225. doi: 10.3389/fmed.2021.748225 34869439 PMC8633540

[8] Bao X Zhang H Wu W Cheng S Dai X Zhu X. Analysis of the molecular nature associated with microsatellite status in colon cancer identifies clinical implications for immunotherapy. J Immunother Cancer (2020) 8(2):e001437. doi: 10.1136/jitc-2020-001437 33028695 PMC7542666

[9] He J Zhao B Huang X Fu X Liu G Tian Y. Gene network analysis reveals candidate genes related with the hair follicle development in sheep. BMC Genomics (2022) 23(1):428. doi: 10.1186/s12864-022-08552-2 35672687 PMC9175362

[10] Zhao Y Zhang Y Dai C Hong K Guo Y. A signature constructed with mitophagy-related genes to predict the prognosis and therapy response for breast cancer. Aging (Albany NY) (2022) 14(15):6169–86. doi: 10.18632/aging.204209 PMC9417220 35939339

[11] Li J Zhang Y Lu T Liang R Wu Z Liu M. Identification of diagnostic genes for both alzheimer's disease and metabolic syndrome by the machine learning algorithm. Front Immunol (2022) 13:1037318. doi: 10.3389/fimmu.2022.1037318 36405716 PMC9667080

[12] Lai Y Lin X Lin C Lin X Chen Z Zhang L. Identification of endoplasmic reticulum stress-associated genes and subtypes for prediction of alzheimer's disease based on interpretable machine learning. Front Pharmacol (2022) 13:975774. doi: 10.3389/fphar.2022.975774 36059957 PMC9438901

[13] Yang B Bao W Wang J. Active disease-related compound identification based on capsule network. Brief Bioinform (2022) 23(1):bbab462. doi: 10.1093/bib/bbab462 35057581 PMC8690041

[14] Bao W Cui Q Chen B Yang B. Phage_UniR_LGBM: phage virion proteins classification with UniRep features and LightGBM model. Comput Math Methods Med (2022) 2022:9470683. doi: 10.1155/2022/9470683 35465015 PMC9033350

[15] Yang B Bao W Wang J Chen B Iwamori N Chen J. Disease-related compound identification based on deeping learning method. Sci Rep (2022) 12(1):20594. doi: 10.1038/s41598-022-24385-1 36446871 PMC9708143

[16] Zhang D Liu J Gao B Zong Y Guan X Zhang F. Immune mechanism of low bone mineral density caused by ankylosing spondylitis based on bioinformatics and machine learning. Front Genet (2022) 13:1054035. doi: 10.3389/fgene.2022.1054035 36468006 PMC9716034

[17] Zhou K Cai C He Y Chen Z. Potential prognostic biomarkers of sudden cardiac death discovered by machine learning [published online ahead of print, 2022 sep 29]. Comput Biol Med (2022) 150:106154. doi: 10.1016/j.compbiomed.2022.106154 36208596

[18] Zhang L Chu XF Xu JW Yao XY Zhang HQ Guo YW. Identification and exploration of the pyroptosis-related molecular subtypes of breast cancer by bioinformatics and machine learning. Am J Transl Res (2022) 14(9):6521–35. doi: 10.1007/s00109-022-02261-9 PMC9556502 36247248

[19] Beck MW. NeuralNetTools: visualization and analysis tools for neural networks. J Stat Software (2018) 85(11):1–20. doi: 10.18637/jss.v085.i11 PMC6262849 30505247

[20] Sun D Peng H Wu Z. Establishment and analysis of a combined diagnostic model of alzheimer's disease with random forest and artificial neural network. Front Aging Neurosci (2022) 14:921906. doi: 10.3389/fnagi.2022.921906 35847663 PMC9280980

[21] Farris AB Colvin RB. Renal interstitial fibrosis: mechanisms and evaluation. Curr Opin Nephrol Hypertens (2012) 21(3):289–300. doi: 10.1097/MNH.0b013e3283521cfa 22449945 PMC3354760

[22] Guo X Adeyanju O Sunil C Mandlem V Olajuyin A Huang S. DOCK2 contributes to pulmonary fibrosis by promoting lung fibroblast to myofibroblast transition. Am J Physiol Cell Physiol (2022) 323(1):C133–44. doi: 10.1152/ajpcell.00067.2022 PMC9273279 35584329

[23] Gu L Li A Lin J Gan Y He C Xiao R. Knockdown of SOX9 alleviates tracheal fibrosis through the wnt/β-catenin signaling pathway. J Mol Med (Berl) (2022) 100(11):1659–70. doi: 10.1007/s00109-022-02261-9 36192639

[24] Scharf GM Kilian K Cordero J Wang Y Grund A Hofmann M. Inactivation of Sox9 in fibroblasts reduces cardiac fibrosis and inflammation. JCI Insight (2019) 5(15):e126721. doi: 10.1172/jci.insight.126721 31310588 PMC6693887

[25] Athwal VS Pritchett J Llewellyn J Martin K Camacho E Raza SM. SOX9 predicts progression toward cirrhosis in patients while its loss protects against liver fibrosis. EMBO Mol Med (2017) 9(12):1696–710. doi: 10.15252/emmm.201707860 PMC5709769 29109128

[26] Ostendorf T Eitner F Floege J. The PDGF family in renal fibrosis. Pediatr Nephrol (2012) 27(7):1041–50. doi: 10.1007/s00467-011-1892-z 21597969

[27] Chen YT Chang FC Wu CF Chou YH Hsu HL Chiang WC. Platelet-derived growth factor receptor signaling activates pericyte-myofibroblast transition in obstructive and post-ischemic kidney fibrosis. Kidney Int (2011) 80(11):1170–81. doi: 10.1038/ki.2011.208 21716259

[28] Duffield JS. Macrophages and immunologic inflammation of the kidney. Semin Nephrol (2010) 30(3):234–54. doi: 10.1016/j.semnephrol.2010.03.003 PMC2922007 20620669

[29] Mehrotra P Collett JA McKinney SD Stevens J Ivancic CM Basile DP. IL-17 mediates neutrophil infiltration and renal fibrosis following recovery from ischemia reperfusion: compensatory role of natural killer cells in athymic rats. Am J Physiol Renal Physiol (2017) 312(3):F385–97. doi: 10.1152/ajprenal.00462.2016 PMC5374313 27852609

[30] Wang Y Li Y Chen Z Yuan Y Su Q Ye K. GSDMD-dependent neutrophil extracellular traps promote macrophage-to-myofibroblast transition and renal fibrosis in obstructive nephropathy. Cell Death Dis (2022) 13(8):693. doi: 10.1038/s41419-022-05138-4 35941120 PMC9360039

[31] Cao Q Wang Y Harris DC. Macrophage heterogeneity, phenotypes, and roles in renal fibrosis. Kidney Int Suppl (2011) (2014) 4(1):16–9. doi: 10.1038/kisup.2014.4 PMC4536959 26312145

[32] Kurusu A Suzuki Y Horikoshi S Shirato I Tomino Y. Relationship between mast cells in the tubulointerstitium and prognosis of patients with IgA nephropathy. Nephron (2001) 89(4):391–7. doi: 10.1159/000046109 11721155

