# Supplementary material for: Construction of a neural network diagnostic model for renal fibrosis and investigation of immune infiltration characteristics
Source: Front Immunol. 2023 Jun 9;14:1183088. doi: 10.3389/fimmu.2023.1183088 (PMC10288286; doi:10.3389/fimmu.2023.1183088)
Supplement: Supplementary file 1 [file DataSheet_1.zip › Tables/Data Sheet 1.zip!/Supplementary materials(tables)/Table 1.doc]

Supplementary Table 1 Key regulators and signaling pathways of ferroptosis in diabetic nephropathy

| Regulators / pathways | Experimental models | Effects | Mechanisms | References |
| --- | --- | --- | --- | --- |
| circASAP2 | C57BL/6 mice treated with a  high- diet and 30mg/kg STZ  HK-2 cells treated with high glucose | Elevated blood glucose;  Deteriorated kidney function;  Aggravated glomerulus injury;  Enhanced oxidative stress;  Exacerbated inflammation;  Promoted ferroptosis. | circASAP2↓, miR-770-5p↑,  IL-1β↑，IL-6↑, INF-γ↑,  TNF-α↑, MDA↑, SOD↓,  CAT↓,GSH-Px↓, GPX4↓,  SOX2↓, SLC7A11↓ | [37] |
| hsa_circ_0006370 | HK-2 cells treated with high glucose | Reduced cell viability;  Enhanced oxidative stress;  Promoted ferroptosis. | hsa_circ_0006370↑ , ROS↑,  MDA↑,LDH↑, GSH-Px↓,  GPX4↓, xCT↓, FT↓, TFR↑,  Fe ^2+^ level↑, Nrf2↓, HO-1↓ | [38] |
| COX-2 | DBA/2J mice treated with 40mg/kg STZ  HK-2 cells treated with high glucose | Elevated blood glucose;  Deteriorated kidney function;  Aggravated renal tubular injury;  Caused mitochondrial damage;  Promoted ferroptosis. | COX-2↑, SLC7A11↓, FTH-1↓, TFR-1↑, GSH↓, MDA↑,  4-HNE↑, iron content↑, PTGS2↑ | [47] |
| ALOX15 | DN patients  C57/J mice treated with 50mg/kg STZ, *db/db* diabetic mice | Promoted ferroptosis. | ALOX15↑, GPX4↓ | [58] |
| Prdx6 | C57BL/6 mice treated with 50mg/kg STZ  MPC5 cells treated with high glucose | Deteriorated kidney function;  Caused podocyte dysfunction;  Enhanced oxidative stress;  Exacerbated inflammation;  Promoted ferroptosis. | Prdx6↓, LDH↑, IL-6↑,  TNF-α↑, ROS↑, MDA↑,  SOD↓, GSH↓, iron↑,  SLC7A11↓, GPX4↓ | [65] |
| ZIP14 | SD rats treated with 60mg/kg  STZ  HK-2 cells treated with high glucose | Elevated blood glucose;  Deteriorated kidney function;  Aggravated renal tubular injury;  Caused mitochondrial damage;  Enhanced oxidative stress;  Promoted ferroptosis. | ZIP14↑, MDA↑, GSH↓,  SOD↓, ROS↑, ferroportin↑,  TFR1↑, Fe^2+^ content↑,  GPX4↓ | [76] |
| HMGB1 | Patients with diabetic kidney disease  SV40-MES 13 cells treated  with high glucose | Enhanced oxidative stress;  Exacerbated inflammation;  Promoted ferroptosis. | HMGB1↑,serum ferritin↑,  LDH↑, ACSL4↑, PTGS2↑,  NOX1↑, GPX4↓, ROS↑,  MDA↑, IL-6↑, TNF-α↑,  TLR4↑, p-NF-κB↑ | [96] |
| Nrf2 | DBA/2J mice treated with  40mg/kg STZ  HK-2 cells treated with high  glucose | Elevated blood glucose;  Increased urine protein;  Deteriorated kidney function;  Aggravated renal pathological damage;  Reduced cell viability;  Enhanced oxidative stress;  Promoted ferroptosis. | Nrf2↓, MDA↑,4-HNE↑,  GSH↓, ROS↑, iron content↑,  FTH-1↓,TFR-1↑GPX4↓,  SLC7A11↓ | [108,109] |
| GPX4 | Patients with diabetic kidney disease | Promoted ferroptosis. | GPX4↓ | [115-117] |
| GPX4 | Beagle treated with STZ and  ALX 20mg/kg  Madin-darby canine kidney  cells terated with high glucose | Elevated blood glucose;  Deteriorated kidney function;  Aggravated renal pathological damage;  Exacerbated mitochondrial oxidative damage;  Destroyed mitochondrial antioxidant system;  Promoted ferroptosis. | GPX4↓, SLC7A11↓, Ferritin↑, MDA↑, H_2_O_2_↑, protein carbonyl↑, 8-ohdg↑, Nos3↑, Mn-SOD2↓,  CAT↓, GSH↓, TRXR↓,  Ac-SOD2↑, Ac-SOD2/SOD↑, total thiol↓, non-protein thiol↓, Prdx3↓, Sirt3↓, Grx3↓, IDH2↑, ROS↑ | [118] |
| HIF-1α/HO-1 pathway | *db/db* diabetic mice | Elevated blood glucose;  Increased urine protein;  Aggravated renal tubular injury；  Exacerbated renal fibrosis；  Enhanced oxidative stress;  Promoted ferroptosis;  Accelerated apoptosis. | HIF-1α↑, HO-1↑,iron ion↑,  ferritin↑, transferrin↑,  iron content↑,ferritin heavy chain↑,  ROS↑,gp91 phox↑, MDA↑,  SOD↓, CAT↓, GSH-Px↓,  GPX4↓,Cleaved caspase-3↑ | [131] |
