# Supplementary material for: Construction of a neural network diagnostic model for renal fibrosis and investigation of immune infiltration characteristics
Source: Front Immunol. 2023 Jun 9;14:1183088. doi: 10.3389/fimmu.2023.1183088 (PMC10288286; doi:10.3389/fimmu.2023.1183088)
Supplement: Supplementary file 1 [file DataSheet_1.zip › Tables/Data Sheet 1.zip!/Supplementary materials(tables)/Table 2.doc]

Supplementary Table 2 The role of Chinese herbal medicine in the regulation of ferroptosis in DN

| Category | Chinese herbal medicine | Experimental models | Effects | Mechanisms | References |
| --- | --- | --- | --- | --- | --- |
| Flavonoids | Glabridin | SD rats treated with high-fat diet and 40mg/kg STZ  NRK-52E cells treated with high glucose | Lowered blood glucose;  Alleviated insulin resistance;  Attenuated pancreatic and renal pathological damage;  Reduced urine protein;  Improved renal function;  Alleviated oxidative stress;  Inhibited ferroptosis. | ROS↓, MDA↓, CAT↑, GSH↑, SOD↑, GPX4↑, SLC7A11↑, SLC3A2↑, TfR1↓,VEGF↓,  iron concentration↓,  p-AKT↓, p-ERK1/2↓ | [136] |
|  | Calycosin | *db/db* diabetic mice  HK-2 cells treated with high glucose | Improved renal function;  Attenuated tubular injury and fibrosis;  Alleviated oxidative stress;  Inhibited ferroptosis. | LDH↓, MDA↓, lipid ROS↓,  GSH↑, GPX4↑, NCOA4↓ | [141] |
|  | Total flavones of *Abelmoschus manihot* | SD rats treated high-fat diet, unilateral nephrectomy, and 35mg/kg STZ  NRK-52E cells treated with  AGEs | Reduced urine protein;  Improved renal function;  Attenuated tubular injury;  Mitigated mitochondrial damage;  Alleviated oxidative stress;  Inhibited ferroptosis. | kidney and serum nonheme iron levels↓, MDA↓, TfR1↓,  ROS↓, GPX4↑, ACSL4↓,  SLC7A11↑ | [147] |
|  | Mangiferin monosodium salt | SD rats treated high-fat diet  and 30mg/kg STZ | Reduced urine protein;  Improved renal function;  Attenuated renal pathological damage;  Attenuated glycolipid metabolic abnormalities;  Alleviated systemic IR;  Ameliorated podocyte IR；  Suppressed renal inflammation;  Alleviated oxidative stress;  Inhibited ferroptosis. | TNF-α↓, IL-6↓, IL-1β↓,  IL-10↑, IL-18↓, NF-κB p65↓，  p-p38 MAPK/p38 MAPK↓, p-JNK/JNK↓, GPX4↑,  ACSL4↓, DMT1↓, FSP1↑,  NOX2/COX IV↓, CoQ10H2/CoQ10↑,4-HNE↓, MDA↓, ROS↓, O2^-^↓, GSH/GSSG↑, CAT↑,  T-SOD↑, p-PI3K/PI3K↑, p-Akt/Akt↑, p-IRS1(Tyr608)/IRS1↑ | [158] |
| Terpenoids | Glycyrrhizic acid | Podocytes treated with high glucose | Alleviated oxidative stress;  Inhibited ferroptosis. | GPX4↑,ROS↓ | [164] |
|  | Ginkgolide B | *db/db* diabetic mice treated with high-fat and high-sugar diet  MPC5 treated with palmitic  acid and high glucose | Lowered blood glucose;  Reduced urine protein;  Improved renal function;  Attenuated renal pathological damage;  Improved lipid metabolism;  Decreased fibrosis;  Alleviated oxidative stress;  Inhibited ferroptosis. | GPX4↑, TfR1↓,FTH1↑,  ROS↓, iron content↓ | [170] |
|  | Germacrone | *db/db* diabetic mice  MPC5 cells treated with high  glucose | Lowered blood glucose;  Reduced urine protein;  Improved renal function;  Attenuated renal pathological damage;  Reduced apoptosis;  Mitigated mitochondrial damage;  Alleviated oxidative stress;  Inhibited ferroptosis. | lipid ROS↓, ROS↓, GPX↑, GSH↑, iron content↓,  COX-2↓, ACSL4↓, NOX1↓,  GPX4↑, FTH1↑ | [177] |
| Anthraquinones | Sennoside A | C56BL/6J mice treated with high-fat diet and 100mg/kg STZ | Reduced urine protein;  Alleviated oxidative stress;  Inhibited ferroptosis. | MDA↓, GSH↑, PTGS2↓,  GPX4↑, Nrf2↓, HO-1↓ | [185] |
| Alkaloids | Berberine | MPC5 cells treated with high glucose | Alleviated oxidative stress;  Mitigated mitochondrial damage;  Inhibited ferroptosis. | Nrf2↑, HO-1↑, GPX4↑,  ACSL4↓, PTGS2↓, ROS↓,  GSH↑ | [201] |
|  | Leonurine | HK-2 cells treated with erastin | Inproved cell viability;  Mitigated mitochondrial damage;  Alleviated oxidative stress;  Inhibited ferroptosis. | Nuclear Nrf2↑,ROS↓, P62↑,  GSH↑, GPX4↑, HO-1↑ | [209] |
| Coumarins | Umbelliferone | *db/db* diabetic mice  HK-2 cells treated with high glucose | Lowered blood glucose;  Reduced urine protein;  Improved renal function;  Attenuated renal pathological damage;  Alleviated oxidative stress;  Inhibited ferroptosis. | ROS↓, GSH↑, MDA↓,  iron content↓,ACSL4↓,  GPX4↑, Nrf2↑, HO-1↑ | [222] |
| Lignans | Schisandrin A | C57BL/6 mice treated with high-fat diet and 30mg/kg STZ  Human renal glomerular endothelial cells treated with high glucose | Lowered blood glucose;  Attenuated glomerulus injury;  Reduced urine protein;  Improved renal function;  Alleviated oxidative stress;  Suppressed renal inflammation;  Reduced pyroptosis;  Inhibited ferroptosis. | IL-6↓, INF-γ↓, TNF-α↓,  IL-1α↓, IL-1β↓, GSDMD↓,  TXNIP↓, NLRP3↓, CAT↑,  Caspase-1↓,MDA↓, SOD↑,  GSH-Px↑, ROS↓, GPX4↑,  iron concentration↓, SOD2↑,  Nrf2↑,HO-1↑,AdipoR1↑, p-AMPK↑ | [224] |
| Polysaccharides | *Polygonatum sibiricum* polysaccharides | SD rats treated with high-fat, high-sugar diet, and 30mg/kg  STZ | Reduced urine protein;  Improved renal function;  Attenuated renal pathological damage;  Alleviated oxidative stress;  Inhibited ferroptosis. | MDA↓, GSH↑, total iron↓,  transferrin↓, FTH1↓, GPX4↑ | [233] |
| Saponins | Platycodin D | HK-2 cells treated with high glucose | Inproved cell viability;  Alleviated oxidative stress;  Inhibited ferroptosis. | LDH↓, MDA↓, lipid ROS↓,  Fe^2+^ content↓,GSH↑, TfR1↓,  FTH1↑,SLC7A11↑, ACSL4↓,  GPX4↑ | [242] |
| Carotenoids | Crocetin | Human glomerular mesangial  cells treated with high glucose | Improved cell survival rate;  Inhibited ferroptosis. | ROS↓, GPX4↑, HO-1↑,  Nrf2↑ | [250] |
